# Characterization of Polyphenolic Compounds in Cantaloupe Melon By-Products

**DOI:** 10.3390/foods8060196

**Published:** 2019-06-06

**Authors:** Filomena Monica Vella, Domenico Cautela, Bruna Laratta

**Affiliations:** 1 Consiglio Nazionale delle Ricerche (CNR), Istituto di Ricerca sugli Ecosistemi Terrestri (IRET), via P. Castellino, 111-80131 Napoli, Italy; monica.vella@iret.cnr.it; 2 Stazione Sperimentale per le Industrie delle Essenze e dei derivati dagli Agrumi (SSEA), Azienda Speciale della Camera di Commercio di Reggio Calabria, via T. Campanella, 12-89125 Reggio Calabria, Italy; dcautela@ssea.it

**Keywords:** *Cucumis melo*, polyphenols, flavonoids, antioxidants, by-products, waste valorization

## Abstract

The Muskmelon (*Cucumis melo* L.), which includes several crops of great economic importance worldwide, belongs to the Cucurbitaceae family, and it is well recognized for culinary and medicinal purposes. The high fruit consumption produces a large quantity of waste materials, such as peels and seeds that are still rich in molecules like polyphenols, carotenoids, and other biologically active components that possess a positive influence on human health and wellness. A sustainable development in agro-food and agro-industry sectors could come through the reutilization and valorization of these wastes, which in turn, could result in reducing their environmental impact. The current study provides a biochemical characterization of cantaloupe by-products, peels and seeds, through evaluating total polyphenols, *ortho*-diphenols, flavonoids, and tannins content. Furthermore, the antioxidant activity was assessed in order to understand potential benefits as natural antioxidants. Overall, the peel extract revealed the highest radical’s scavenging and reducing activities, moreover, it showed higher polyphenolic content than seed extract as revealed by both cromatographic and spectrophotometric analyses. The results of the present study indicate that the melon residues are a good source of natural phytochemicals useful for many purposes, such as ingredients for nutraceutic, cosmetic, or pharmaceutical industries, development of functional ingredients and new foods, and production of fertilizers and animal feed.

## 1. Introduction

The Cucurbitaceae family covers several species of great economic importance, including muskmelon (*Cucumis melo* L.), which is largely cultivated and consumed in Europe. Muskmelon encircles a wealth of varietal types, such as smooth-skinned varieties like Honeydew, Crenshaw, and Casaba (*C. melo* var. *inodorous*), rough-skinned varieties like Cantaloupe, Persian melon, and Santa Claus or Christmas melon (*C. melo* var. *reticulatus*), and varieties used when they are immature as vegetables like Barattiere, Carosello, and Armenian Cucumber (*C. melo* var. *flexuosus*). The Cantaloupe melon is well recognized by its net-like slightly ribbed, gray-to-green or light brown skin. It is one of the most consumed melons worldwide thanks to its sweetness, juicy taste, pleasing flavor, and nutritional value [1,2]. In 2016, about 1.9 million tons of melon were harvested in the Mediterranean area, with Spain, Italy, and France representing the main European producers, accounting for 35%, 34%, and 13% overall yield, respectively [3]. In Italy, Cantaloupe is the most cultivated variety. Its name is supposed to derive from Italian “Cantalupo in Sabina”, which was formerly a papal county seat near Rome [4].

Cantaloupe is an excellent source of vitamin A, vitamin C, and microelements such as potassium and magnesium [1,4,5]. In recent years, it has been shown to possess useful medicinal properties such as analgesic, anti-inflammatory, antioxidant, antiulcer, anticancer, antimicrobial, diuretic, and antidiabetic properties [2,6,7]. Furthermore, it showed a hepato-protective effect, activity against hypothyroidism and immune-modulator action [6].

Ever-increasing demand for healthy food has stimulated the manufacturing sectors to search for new natural sources of nutritional and healthful components to be employed as food additives or supplements, with high nutritional value [8,9]. As a consequence, the European Union has encouraged the exploitation of fruit by-products for their use as a source of nutritionally and therapeutically functional ingredients to utilize for dietary intake, and as active ingredients in pharmaceuticals and cosmetic industries [10,11].

During fresh consumption and industrial processing of melons (juices, compotes, and salads), large quantities of peels and seeds are produced, and are considered waste. The complete utilization of these by-products could minimize the litter volume, so reducing the environmental impact and the economic costs associated to their disposal. Peels and seeds, in fact, are potential sources of phytochemicals, such as polyphenols, carotenoids, flavonoids, and other bioactive compounds with potential health-promoting effects [12,13]. Among them, polyphenol compounds show antioxidant activity, delaying or inhibiting the oxidation of lipids and other molecules, thus playing an important role in defending cells against free radical damage, a very important way of preventing diseases like cancer and cardiovascular disorders [12,13,14,15].

Studies related to melon peels and seeds are scarce; data concerning their whole biochemical characterization are insufficient. Recently, Mallek-Ayadi et al. [5] studied only the phenolic composition and functional properties of peels, concluding that this by-product could be considered as a rich source of carbohydrates, proteins, calcium, potassium, and polyphenols. Fundo et al. [1] characterized the edible and the waste parts of cantaloupe melon, only considering the bioactive compounds showing antioxidant activity. These studies suggested that the valorization of cantaloupe by-products should be encouraged because they are important sources of healthy compounds for food, cosmetics, and nutraceutical products. 

On this basis, this research aims to examine the peels and seeds from cantaloupe melon, evaluating total polyphenol, *ortho*-diphenol, flavonoid, and tannin contents. At the best of our knowledge, this is the first time that such a comprehensive investigation, by means of spectrophotometric, chromatographic and in vitro assays, is achieved on both extracts from seeds and peels, in order to explore their potential attitude as natural sources of antioxidants.

## 2. Materials and Methods

### 2.1. Reagents and Standards

Sodium carbonate, Folin–Ciocalteu reagent, sodium nitrite, sodium molybdate, aluminum chloride 6-hydrate, 2,2-diphenyl-1-picrylhydrazyl (DPPH), sodium acetate, iron(III) chloride 6-hydrate, 2,4,6-tripyridyl-s-triazine (TPTZ), bovin serum albumin (BSA), standards phenolic acids (gallic, caffeic, chlorogenic, syringic, ferulic, and ellagic acids), flavonoids (rutin, quercetin, kaempferol and isorhamnetin) and the HPLC-grade solvents were purchased from Sigma Chemical Co. (St. Louis, MO, USA). Sodium hydroxide, hydrochloric acid and ethanol were obtained from Carlo Erba Reagents (Milan, Italy).

### 2.2. Extraction of Bioactive Compounds 

Three “Prescott” varieties of cantaloupe melons (*Cucumis melo* L. var. *reticulatus*) were purchased in a local market at commercial ripening stage. Peels were manually removed with a knife and seeds were separated too. Peels and seeds were oven-dried at 37 °C, until constant weight. Randomly chosen samples were utilized for analyses. Both samples were milled with a food mixer (Moulinex, Italy) and kept at −20 °C until extractions were performed. For bioactive compounds recovery, 200 mg of fine powder of cantaloupe peels and seeds were extracted with 10 mL of 95% ethanol (ratio 1:50 w/v) for 6 h at 50 °C in a closed vessel using an ultrasonic bath. The extracts were recovered by centrifugation at 13,000× *g* for 10 min at 4 °C, and dried using a rotary evaporator (IKA RV8, IKA-Werke GmbH & Co, Staufen, Germany). 

### 2.3. Total Polyphenols Content

Total polyphenols were spectrophotometrically determined according to the Folin–Ciocalteu method [16]. In brief, 150 µL of extracts were mixed with 750 µL of Folin–Ciocalteu reagent and 600 µL of 7.5% (*w*/*v*) Na_2_CO_3_. After incubation, the absorbance was read at 765 nm (UV-Vis spectrophotometer, model DMS-200, Varian, Leini, Italy). Total phenolic amount was calculated by a six point calibration curve obtained with different quantities of gallic acid standard solution ranging from 1.5 to 10 µg. (y = 0.0768 x; R^2^ = 0.9909) and the results were expressed as mg of gallic acid equivalents (GAE) per g of extract.

### 2.4. Ortho-Diphenols Content

*Ortho*-diphenols content was evaluated by Arnow assay [17]. Briefly, 400 µL of extracts were mixed with 400 µL of 0.5 M HCl, 400 µL of 1.45 M NaNO_2_—0.4 M Na_2_MoO_4_ and 400 µL of 1 M NaOH. The absorbance was recorded at 500 nm and *ortho*-diphenols were determined by a calibration curve obtained using caffeic acid as standard. The *ortho*-diphenolic content was determined by a calibration curve obtained using a caffeic acid standard solution ranging from 5 to 50 µg (y = 0.0152 x; R^2^ = 0.9921), and the results were expressed as mg of caffeic acid equivalents (CAE) per g of extract.

### 2.5. Flavonoid Content

Flavonoid content in the extracts was determined according to the colorimetric method based on the formation of flavonoid-aluminum compounds [18]. In the assay, extracts were mixed with distilled water and NaNO_2_. After 5 min, AlCl_3_ x 6H_2_O were added and the reaction was stopped by adding 1 M NaOH and distilled water. The absorbance was read at 510 nm and (+)-catechin was used to create the standard curve. (+)-Catechin, from 5 to 100 μg was used to create the calibration curve (y = 0.009 x; R^2^ = 0.9940) and the results were expressed as mg of catechin equivalents (CE) per g of extract.

### 2.6. Tannins Content

Total tannins were assessed as reported by Vella et al. [19] incubating extracts with BSA at 30 °C for 1 h. The supernatant, representing the non-tannin fraction, was collected by centrifugation at 13,000× *g* for 10 min at 4 °C and was analyzed using the Folin-Ciocalteu method. Tannins were determined by difference from the amounts of the polyphenols determined before and after BSA precipitation. Tannins were expressed as mg of gallic acid equivalents (GAE) per g of extract.

### 2.7. In Vitro Antioxidant Activity

The antioxidant activity of cantaloupe peels and seed extracts were evaluated by means of two in vitro biochemical assays: The Ferric Reducing Antioxidant Power (FRAP) and the DPPH (2,2-diphenyl-1-picrylhydrazyl) radical-scavenging activity. 

As reported by Benzie and Strain [20], freshly prepared FRAP reagent were added to the extracts. The absorbance was recorded after 4 min at 593 nm. The antioxidant activity of samples was calculated from a calibration curve with L-ascorbic acid ranging from 0.5 to 5 μg (y = 0.1662 x; R^2^ = 0.9918) and the results were expressed as mg of ascorbic acid equivalents (AAE) per g of extract. 

The free radical scavenging activity (RSA) of the extracts was assessed according to the procedure of Blois [21]. In brief, different concentrations of peels and seeds extracts were mixed with DPPH methanolic solution. The absorbance reduction at 517 nm of the DPPH was determined continuously. The RSA was calculated as a percentage of DPPH discoloration, using the following equation:

(1) $$\%\;RSA=\;\left[\frac{\left(A_{DPPH}-A_{s}\right)}{A_{DPPH}}\right]*100,$$

where A_S_ is the absorbance of the solution when the extract was added and A_DPPH_ is the absorbance of the DPPH solution. The EC_50_ value was obtained from the graph of %RSA against the extract concentrations in mg/mL.

### 2.8. Cromatographic Analyses

High performance liquid chromatography-photodiode-array-mass spectrometry (HPLC-PDA-ESI-MS/MS) analyses were performed using a Surveyor LC pump, a Surveyor autosampler, coupled with a photodiode array detector (PDA) Surveyor and a LCQ Advantage ion trap mass spectrometer (Thermo Finnigan, Waltham, MA, USA) equipped with Xcalibur 3.1 software (Thermo Fisher Scientific, Waltham, MA, USA). 

A volume of 5 μL was employed for the analysis on a Supelco Spherisorb^®^ ODS2 HPLC Column (250 × 4.6 mm), and the column was thermostatically controlled at 35 °C. The elution was conducted, as already reported [22], by employing 0.3% acetic acid solution (solvent A) and acetonitrile (solvent B). A gradient elution was performed as following: the initial solvent was 90% A and 10% B; the gradient elution was changed from 10% to 20% B in a linear mode for 15 min; this composition was maintained at isocratic flow for 10 min; the solvent B reached 50% in 10 min and from 50 to 90% B in 10 min. 

Elution was performed at a flow rate of 0.5 mL/min with a splitting system of 2:8 to the MS detector (100 μL/min) and PDA detector (400 μL/min). Analyses were performed with an electrospray ionization (ESI) interface in the negative mode. The optimization of the instrumental parameters for bioactive compounds was performed by continuous infusion (FIA)-ESI MS/MS analyses. Parameters for analysis were set using negative and positive ion modes, with spectra acquired over a mass range from *m*/*z* 50 to 1100. The ionization conditions were optimized, and the parameters used were as follows: capillary temperature, 210 °C; capillary voltage, −10.0 V; tube lens offset, −50.0 V; sheath gas flow rate, 60.00 arbitrary units; auxiliary gas flow rate, 20.00 arbitrary units; spray voltage, 4.50 kV; and scan range of *m*/*z* 150–1200. In the MS/MS experiments, normalized collision energy of 35.0% was applied. 

PDA data were recorded with 200–600 nm range, and HPLC/UV chromatograms were acquired at three different wavelengths (226, 284 and 369 nm) according to the absorption maxima of analyzed compounds. Figure 3 shows the 284 nm absorption data for all compounds. For the quantitative analysis of phenolic compounds, a calibration curve was obtained by the injection of different concentrations of each standard. Peak identification of phenolic compounds was performed according to their retention time, UV-Vis and mass spectra.

For the quantification of phenolic compounds by HPLC-UV, a calibration curve was obtained through injection of different concentrations of standard mixture, blending aliquots of different stock individual standards into a 10 mL glass volumetric flask. The standard solutions were prepared in methanol in the range of 0.0025–0.045 mg/mL. The reproducibility of the detector response at each concentration level was evaluated by a triplicate injection of standard mix and expressed as percentage of relative standard deviations (RSD%). The RSDs were expected to be less than 2%. The limits of detection (LOD) were established at a signal to noise ratio (S/N) of 3. The limits of quantification (LOQ) were established at a signal to noise ratio (S/N) of 10. LOD and LOQ were experimentally verified by the nine injections of reference compounds in LOQ concentrations.

### 2.9. Statistical Analysis

All samples were analyzed in triplicates and the results were expressed as mean ± standard deviation (SD). Means, SD, calibration curves and linear regression analyses (R^2^) were determined using Microsoft Excel 2013 (Microsoft Corporation, Redmond, WA, USA). 

## 3. Results and Discussion

The phenolic composition and functional activity of melon residues, peels and seeds, were studied in order to explore their beneficial properties in sight of potential industrial applications. In this contribution, a biochemical characterization was obtained through the evaluation of total polyphenols, *ortho*-diphenols, flavonoids, tannins, and antioxidants by means of photometric assays and by HPLC profiling. 

**Figure 1:**
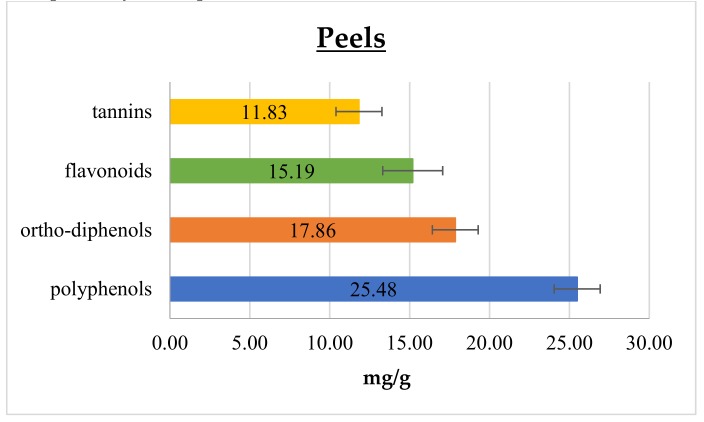
Polyphenol, *ortho*-diphenol, flavonoid, and tannin contents in cantaloupe peels.

**Figure 2:**
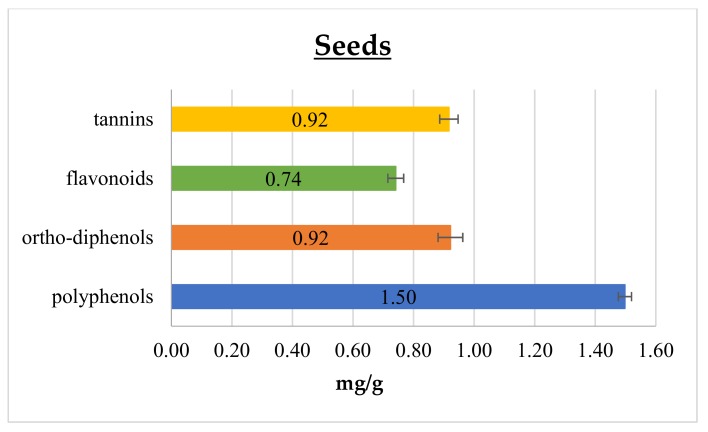
Polyphenol, *ortho*-diphenol, flavonoid, and tannin contents in cantaloupe seeds.

In Figure 1 and Figure 2 polyphenol, *ortho*-diphenol, flavonoid, and tannin contents in cantaloupe peels and seeds, respectively, are reported. 

In peels, the polyphenol content was 25.48 ± 1.44 mg GAE/g, which is 6- and 8-fold higher than that reported by Isamil et al. [2] and Mallek-Ayadi et al. [5], respectively. This gap could be attributed to several factors, including cultivar, degree of ripening, and environmental elements, such as climatic conditions and geographical origin [4,19,22]. Conversely, cantaloupe seeds content of polyphenols was 1.50 ± 0.02 mg GAE/g, and this result matches with the range reported in literature data [1,2]. Polyphenols are commonly found in both edible and non-edible plant parts, being essential compounds for their growth and reproduction pathways. They also play an important role in modulating the defense response against insects, pathogens, and microorganisms. Moreover, they take an active part in determining color, flavor, taste, and appearance of fruits. Among phenolics, *ortho*-diphenols are recognized as the most important in relation to their antioxidant activity, since they are able to improve radical stability by forming an intra-molecular hydrogen bond between the hydrogen and phenoxyl radicals. As reported in Figure 1 and Figure 2, *ortho*-diphenols content was 17.86 ± 1.43 and 0.92 ± 0.04 mg CAE/ g in peels and seeds, respectively. To the best of our knowledge, data on *ortho*-diphenols have never been reported in cantaloupe until now.

Considering flavonoids, they are the most common and widely distributed group of plant phenolics, being very effective antioxidants [23]. Cantaloupe peels showed the highest flavonoid content of 15.19 ± 1.88 mg CE/g, meanwhile seeds had 0.74 ± 0.03 mg CE/g, as shown in Figure 1 and Figure 2, respectively. In addition, these components scored higher content values, as well for the total phenolic compounds, than those reported in the literature [2,5]. Moreover, similarly to polyphenols, flavonoid content has many sources of variation such as genotype, fruit ripening, plant phenotypic state and pedoclimatic conditions [4,19,22].

Tannins have been considered health-promoting components of plants, since possessing anti-carcinogenic and anti-mutagenic potentials, as well as antimicrobial, antioxidant and antiradical properties [24,25,26,27]. In the present study, tannins content was higher in peels than in seeds, displaying 11.83 ± 1.44 and 0.92 ± 0.03 mg GAE/g, respectively. The literature reports many studies comparing polyphenolic and flavonoid contents of different parts of cantaloupe [1,2,5] but tannins were never assessed. 

As the phenolic compounds are known to protect cellular components against free radicals, the antioxidant properties of cantaloupe peels and seed extracts were estimated by means of the FRAP assay and by the DPPH radical scavenging activity, whose results are shown in Table 1. As reported by Benzie and Strain [20], the FRAP assay measures the reduction of a ferric 2,4,6-tripyridyl-s-triazine complex (Fe^3+^-TPTZ) to the ferrous form (Fe^2+^-TPTZ) in the presence of an antioxidant compound. The antioxidant ability, measured by the FRAP assay, indicated a higher value in peels as compared to seeds, with values of 12.27 ± 1.22 mg AAE/g and 0.31 ± 0.02 mg AAE/g, respectively. 

The scavenging activity was studied by means of the DPPH assay, based on the evaluation of the reduction of the DPPH radical to hydrazine as a consequence of the antiradical activity of the extracts. Similarly to the results of the FRAP assay, scavenging activity in the peels extracts was stronger since the EC_50_ after 15 min was 6.65 mg/mL, while seed extracts showed a value of 55.03 mg/mL, thus indicating a lower antioxidant activity of this latter cantaloupe by-product. These results were in agreeance with literature data: in particular, the cantaloupe peels extract in our experiment proved to be 1.4 fold more active when compared to the results published by Isamil et al. [2]. Conversely, seed extract showed an activity that was half than that observed and reported by Isamil et al. [2]. The results of the DPPH assay suggest that extracts are capable of scavenging free radicals via electron or hydrogen-donating mechanisms. Moreover, DPPH activity of these cantaloupe by-products showed similar behavior with the polyphenols, *ortho*-diphenols, flavonoids, and tannins content, thus indicating that radical scavenging activity of cantaloupe peels and seeds extracts is related to the amount of phenolic compounds.

Nowadays, synthetic antioxidants are widely used as additives in food, pharmaceuticals, and cosmetics, but their uses have been questioned because of their possible toxic or carcinogenic activities due to some components formed during their degradation occurring in industrial processing [28]. Therefore, the application of natural plant-based substances may be a suitable alternatives to replace artificial molecules, not only because of their safety, but also since they protect food, feed, and derivatives from the deleterious effects of natural oxidation.

**Figure 3:**
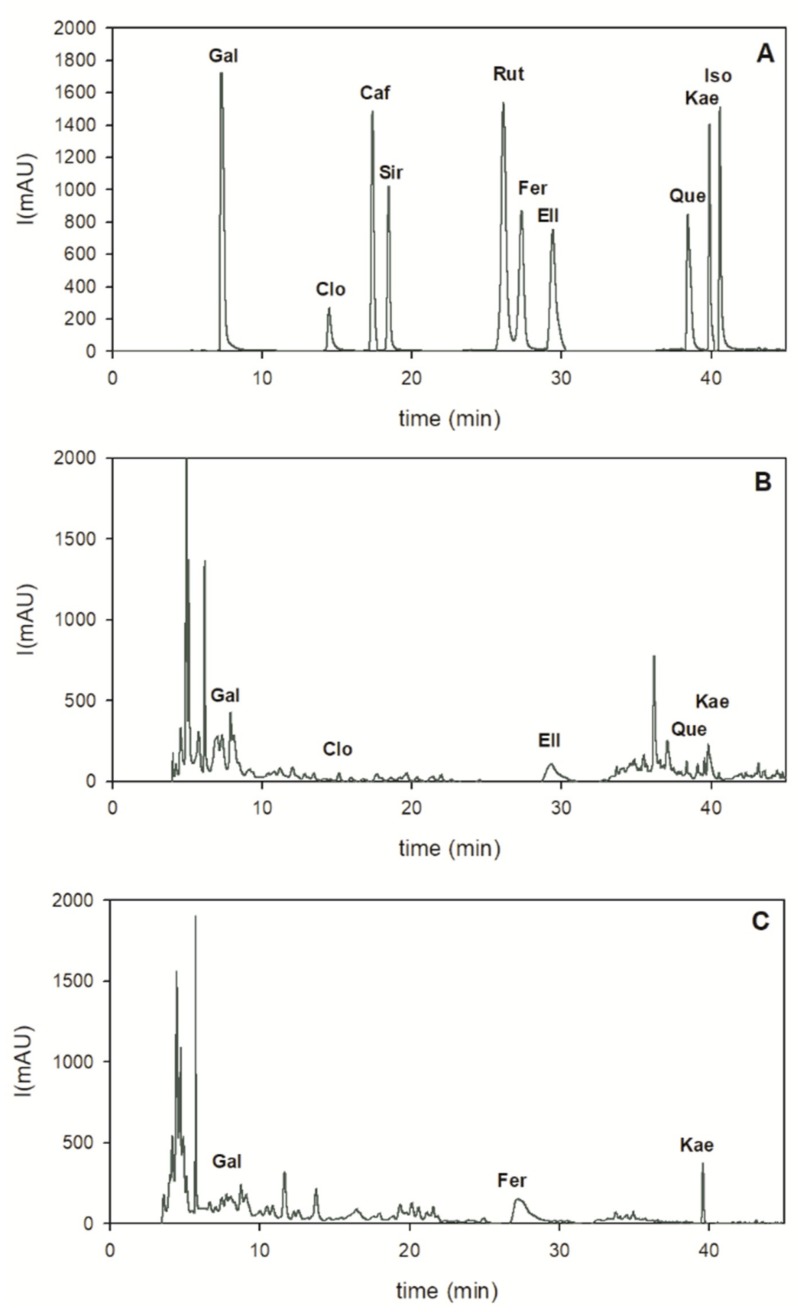
HPLC chromatograms of melon peel and seed extracts, monitored at 284 nm. (**A**) Standard mixture of bioactive compounds. (**B**) Melon peels ethanolic extract. (**C**) Melon seeds ethanolic extract. Gallic acid (Gal), Chlorogenic acid (Clo), Caffeic acid (Caf), Syringic acid (Sir), Rutin (Rut), Ferulic acid (Fer), Ellagic acid (Ell), Quercetin (Que), Kaempferol (Kae), Isorhamnetin (Iso).

The two extracts from melon peels and seed were also analyzed by HPLC to evaluate the real composition of the phenolic profiles (Figure 3).

Phenolic acids and flavonoids were quantified according to HPLC-PDA data recorded at 284 nm, where gallic acid (2.45 ± 0.08 mg/g), ellagic acid (0.57 ± 0.01 mg/g), and kaempferol (0.32 ± 0.03 mg/g) were the main bioactive compounds found in the peels extract (Table 2; Figure 3B). In seed extract, the richest phytochemicals were ferulic acid (1.51 ± 0.02 mg/g), followed by kaempferol (0.54 ± 0.02 mg/g), and gallic acid (0.07 ± 0.02 mg/g), as reported in Table 2 and in Figure 3C. Peak identification of phenolic compounds was performed according to their retention time, UV-Vis and mass spectra (Table 2).

Gallic acid, ellagic acid and kaempferol have been reported to have antiviral, anti-mutagenic, anticancer, antioxidant and cytotoxic effects [29,30,31]. Ferulic acid is a ubiquitous natural phenolic compound in seeds; it exhibits a wide variety of biological activities such as antioxidant, anti-inflammatory, antimicrobial, antiallergic, hepatoprotective, anticarcinogenic, antithrombotic, antiviral and vasodilatory actions, increase sperm viability, metal chelation, modulation of enzyme activity, activation of transcriptional factor [31]. Peel and seed HPLC profiles showed some differences from what was reported by Mallek-Ayadi et al. [5] and Zeb [32]. We suppose that these differences might be due to variation relating to different cultivar, environmental conditions during plant growth and fruiting, plant phenotypic state, and possibly due to extraction conditions too [4,19,22].

Altogether, HPLC analysis confirms a higher level of phenolic acids and flavonoids in melon peel than in seed extract. The diverse tissue distribution of bioactive compounds may be attributed to different metabolic roles and networks active in peels and seeds due to their different roles in plant architecture and physiology. Furthermore, it is important to underline that in this study ethanol was used for chemical extraction of peels and seeds and, since it is a GRAS (Generally Recognized As Safe) solvent, it can be utilized safely for bioactive compound recovery, to be used in the food industry.

## 4. Conclusions

Normally, the non-edible parts of the melon (seeds and peels) are discarded during production processes, reaching approximately 8 to 20 million tons of waste per year worldwide [33]. The extracts of melon exhibit valuable functional and nutraceutical properties, in the light of all the data, spectrophotometry, HPLC profiling and biological activity, obtained in the course of the present study.

With the aim of developing new nutraceuticals, such as supplements, dietary and nutritional products, these cantaloupe by-products seem to be very promising, opening up new perspectives for their use, mainly due to the solubility in water and the stability of their extracts. Therefore, the melon extracts could be used in the production of functional waters, greatly demanded by markets and consumers all over the world, or in food and cosmetic products. Indeed, it has been observed that these by-products act against the oxidation process, thus suggesting their possible future uses as natural colorants and antioxidants in yogurt, biscuits, cupcakes, jellies, sweets and bread, and in anti-wrinkle creams, soaps and bathroom foams, as reported in the literature [33]. Moreover, in this study the reduction to a minimum of the waste volumes and the possibility of developing new products, with the recovery of biomolecules with high added value, may contribute to the sustainable management of waste biomasses that otherwise imply environmental and economic costs.

## Figures and Tables

**Table 1:** Antioxidants content and activity in cantaloupe peels and seeds.

	Antioxidant Power (mg AAE/g) *	EC_50_ (mg/mL) **
Peels	12.27 ± 1.22	6.65
Seed	0.31 ± 0.02	55.03

* The antioxidant power was measured by FRAP assay; ** EC_50_ was calculated by DPPH assay.

**Table 2:** Bio-active contents in melon peel and seed extracts (UV-Vis, HPLC and MS data).

Bioactive Compound	Retention Time (min)	Parent Ion [M-H]-(*m*/*z*)	Transitions (*m*/*z*)	Peel Extract (mg/g)	Seed Extract (mg/g)	λ(_max_)
Gallic acid (Gal)	7.39	169.0	125.1	2.45 ± 0.08	0.07 ± 0.02	272
Chlorogenic acid (Clo)	14.62	353.0	191.1	0.08 ± 0.03	0.03 ± 0.01	243,328
Caffeic acid (Caf)	17.52	179.0	135.1	<LOD	<LOD	287,324
Syringic acid (Sir)	18.33	198.1	153.1	<LOD	<LOD	276
Rutin (Rut)	26.15	609.0	299.9	0.06 ± 0.01	nd	258,356
Ferulic acid (Fer)	27.58	193.0	176.9	0.09 ± 0.01	1.51 ± 0.02	287,312
Ellagic acid (Ell)	29.86	301.3	229.0	0.57 ± 0.01	nd	254,364
Quercetin (Que)	38.61	301.0	150.9	0.02 ± 0.01	nd	255,372
Kaempferol (Kae)	39.96	285.0	185.0	0.32 ± 0.03	0.54 ± 0.02	266,366
Isorhamnetin (Iso)	40.51	315.0	286.0	<LOD	<LOD	268,342

LOD = limit of detection S/N: 3 (*n* = 9) LOD = 0.02 μg/mL.

## References

[1] Fundo J.F., Miller F.A., Garcia E., Santos J.R., Silva C.L., Brandão T.R. (2018). Physicochemical characteristics, bioactive compounds and antioxidant activity in juice, pulp, peel and seeds of Cantaloupe melon. J. Food Meas. Charact..

[2] Ismail H.I., Chan K.W., Mariod A.A., Ismail M. (2010). Phenolic content and antioxidant activity of cantaloupe (*Cucumis melo*) methanolic extracts. Food Chem..

[3] Faostat, Food and Agriculture Organization of the United States. http://www.fao.org/home/en/.

[4] Maietti A., Tedeschi P., Stagno C., Bordiga M., Travaglia F., Locatelli M., Arlorio M., Brandolini V. (2012). Analytical traceability of melon (*Cucumis melo* var *reticulatus*): Proximate composition, bioactive compounds, and antioxidant capacity in relation to cultivar, plant physiology state, and seasonal variability. J. Food Sci..

[5] Mallek-Ayadi S., Bahloul N., Kechaou N. (2017). Characterization; phenolic compounds and functional properties of *Cucumis melo* L. peels. Food Chem..

[6] Milind P., Kulwant S. (2011). Muskmelon is eat-must melon. Int. Res. J. Pharm..

[7] Vouldoukis I., Lacan D., Kamate C., Coste P., Calenda A., Mazier D., Conti M., Dugas B. (2004). Antioxidant and anti-inflammatory properties of a *Cucumis melo* LC. extract rich in superoxide dismutase activity. J. Ethnopharmacol..

[8] Galanakis C.M. (2012). Recovery of high added-value components from food wastes: Conventional, emerging technologies and commercialized applications. Trends Food Sci. Technol..

[9] Schieber A., Stintzing F.C., Carle R. (2001). By-products of plant food processing as a source of functional compounds—Recent developments. Trends Food Sci. Technol..

[10] Kosseva M.R. (2009). Processing of food wastes. Adv. Food Nutr. Res..

[11] Laufenberg G., Kunz B., Nystroem M. (2003). Transformation of vegetable waste into value added products: (A) the upgrading concept; (B) practical implementations. Bioresour. Technol..

[12] Dai J., Mumper R.J. (2010). Plant phenolics: Extraction, analysis and their antioxidant and anticancer properties. Molecules.

[13] Moon J.K., Shibamoto T. (2009). Antioxidant assays for plant and food components. J. Agric. Food Chem..

[14] Shahidi F., Naczk M. (2004). Phenolics in Food and Nutraceuticals.

[15] Sroka Z., Cisowski W. (2003). Hydrogen peroxide scavenging, antioxidant and anti-radical activity of some phenolic acids. Food Chem. Toxicol..

[16] Singleton V.L., Rossi J.A. (1965). Colorimetry of total phenolics with phosphomolybdic-phosphotungstic acid reagents. Am. J. Enol. Vitic..

[17] Arnow L.E. (1937). Colorimetric determination of the components of 3,4-dihydroxyphenylalaninetyrosine mixtures. J. Biol. Chem..

[18] Zhishen J., Mengcheng T., Jianming W. (1999). The determination of flavonoids contents in mulberry and their scavenging effects on superoxide radicals. Food Chem..

[19] Vella F.M., Laratta B., La Cara F., Morana A. (2018). Recovery of bioactive molecules from chestnut (*Castanea sativa* Mill.) by-products through extraction by different solvents. Nat. Prod. Res..

[20] Benzie I.F., Strain J.J. (1996). The ferric reducing ability of plasma (FRAP) as a measure of “antioxidant power”: The FRAP assay. Anal. Biochem..

[21] Blois M.S. (1958). Antioxidant determination by the use of a stable free radical. Nature.

[22] Cautela D., Laratta B., Santelli F., Trifirò A., Servillo L., Castaldo D. (2008). Estimating bergamot juice adulteration of lemon juice by high-performance liquid chromatography (HPLC) analysis of flavanone glycosides. J. Agric. Food Chem..

[23] Tadmor Y., Burger J., Yaakov I., Feder A., Libhaber S.E., Portnoy V., Meir A., Tzuri G., Sa’ar U., Rogachev I. (2010). Genetics of flavonoid, carotenoid, and chlorophyll pigments in melon fruit rinds. J. Agric. Food Chem..

[24] Buzzini P., Arapitsas P., Goretti M., Branda E., Turchetti B., Pinelli P., Ieri F., Romani A. (2008). Antimicrobial and antiviral activity of hydrolysable tannins. Mini-Rev. Med. Chem..

[25] Koleckar V., Kubikova K., Rehakova Z., Kuca K., Jun D., Jahodar L., Opletal L. (2008). Condensed and hydrolysable tannins as antioxidants influencing the health. Mini-Rev. Med. Chem..

[26] Santos-Buelga C., Scalbert A. (2000). Proanthocyanidins and tannin-like compounds-nature, occurrence, dietary intake, and effects on nutrition and health. J. Sci. Food Agric..

[27] Rechner A.R., Kuhnle G., Bremner P., Hubbard G.P., Moore K.P., Rice-Evans C.A. (2002). The metabolic fate of dietary polyphenols in humans. Free Radic. Biol. Med..

[28] Chen A.Y., Chen Y.C. (2013). A review of the dietary flavonoid, kaempferol on human health and cancer chemoprevention. Food Chem..

[29] Manach C., Williamson G., Morand C., Scalbert A., Remesy C. (2005). Bioavailability and bioefficacy of polyphenols in humans. I Review of 97 bioavailability studies. Am. J. Clin. Nutr..

[30] Tomas-Barberan F.A., Clifford M.N. (2000). Dietary hydroxybenzoic acid derivates—Nature, occurrence and dietary burden. J. Sci. Food Agric..

[31] Kumar N., Pruthi V. (2014). Potential applications of ferulic acid from natural sources. Biotechnol. Rep. (Amst.).

[32] Zeb A. (2016). Phenolic profile and antioxidant activity of melon (*Cucumis melo* L.) seeds from Pakistan. Foods.

[33] Rolim P.M., Seabra L.M.A.J., de Macedo G.R. (2019). Melon by-products: Biopotential in human health and food processing. Food Rev. Int..

